# When the Eye Reveals Disseminated Tuberculosis: A Pediatric Case of Endophthalmitis With Severe CNS Involvement

**DOI:** 10.1155/crop/8141797

**Published:** 2026-08-06

**Authors:** Nicola De Santi, Laura Curci, Eduardo Bianchi, Barbara Iaccheri, Carlo Pallotto, Daniele Rosignoli, Tito Fiore, Giuseppe Vittorio Luigi De Socio, Carlo Cagini

**Affiliations:** ^1^ Department of Medicine Surgical, Section of Ophthalmology, University of Perugia, S. Maria della Misericordia Hospital, Perugia, Italy, unipg.it; ^2^ Infectious Diseases Clinic, Santa Maria Della Misericordia Hospital, Department of Medicine, University of Perugia, Perugia, Italy, unipg.it

**Keywords:** central nervous system tuberculosis, cerebral tuberculoma, extrapulmonary tuberculosis, ocular tuberculosis, panuveitis, pediatric tuberculosis, tuberculosis, tuberculous endophthalmitis

## Abstract

**Purpose:**

This study aims to report a case of tuberculosis involving central nervous system (CNS) discovered thanks to tubercular endophathlmitis.

**Methods:**

This is a case report.

**Observation:**

A 14‐year‐old male presented with acute visual loss, ocular pain, and redness of the right eye following a recent stay in India. The clinical history was notable for prolonged fever, night sweats, and headache. Ophthalmological examination revealed severe intraocular inflammation consistent with panuveitis and endophthalmitis. Neuroimaging demonstrated a large ring‐enhancing lesion in the right hemisphere with significant mass effect. Initial microbiological investigations, including vitreous sampling, were negative. A positive interferon‐gamma release assay and radiological findings raised suspicion for disseminated tuberculosis, and *Mycobacterium tuberculosis* DNA was subsequently detected in vitreous samples after repeating a second vitrectomy. Antitubercular therapy was started and definitive diagnosis was made with biopsy of the brain lesion, which demonstrated positive cultures for drug‐sensitive *M. tuberculosis*. The clinical course was complicated by retinal detachment, paradoxical enlargement of the cerebral tuberculoma, and postoperative neurological deficits requiring surgical intervention and rehabilitation.

**Conclusion:**

This case highlights the diagnostic complexity of ocular and CNS tuberculosis and emphasizes the need for a high index of suspicion, repeated targeted sampling, and multidisciplinary management to achieve timely diagnosis and appropriate treatment.

## 1. Introduction

Tuberculosis (TB) is still today one of the world’s leading causes of death. More than 10 million people have a diagnosis of TB, with an overall mortality of 1.25 million worldwide in 2023 [1]. After the years of the COVID‐19 pandemic, TB once again became the leading cause of death per single infectious agent [1]. *Mycobacterium tuberculosis,* the infectious agent of TB, has increased propensity for tissues with high oxygen tension; hence, the lungs are the main tissue involved. The infection can however expand to extrapulmonary sites, more easily in immunocompromised patients [2, 3].

In literature, ocular involvement is estimated in a range between 2% and 18% of extrapulmonary forms [4]. Main ocular manifestations is uveitis, such as Anterior Uveitis, panuveitis, multifocal choroiditis, serpiginous‐like choroiditis, and chorioretinitis, followed by retinal vasculitis, optic neuropathy, keratitis, and keratoconjunctivitis [5–12]. Endophthalmitis and panophthalmitis are more infrequent, with a rapid progression until a complete destruction of ocular tissue and a possible eye perforation [5, 9–11].

We reported a young patient affected by endophthalmitis and severe central nervous system (CNS) involvement.

## 2. Case Presentation

We present the case of a 14‐year‐old patient with Indian parents (from the Punjabi region) who was admitted to the ophthalmology ward at Santa Maria della Misericordia Hospital in Perugia on September 7th, 2024, experiencing burning, redness, and vision loss of the right eye (OD) for 7 days.

**Figure 1:**
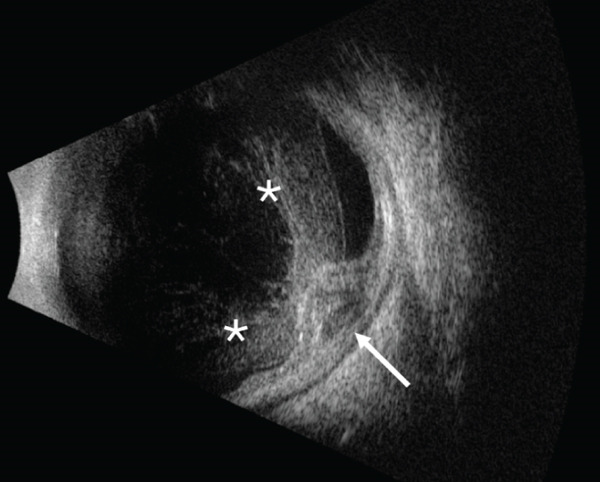
The B‐scan ocular ultrasound imaging showed hyper‐reflective material in vitreous chamber (asterisks) and subretinal mass with high nonhomogeneous echogenicity projecting into the vitreous (arrow).

He went to India to visit his relatives 45 days before, living with other eight people during his stay. At the beginning of August, he developed fever (body temperature > 38°C) with night sweating and fronto‐occipital headache that lasted for 15 days. Fever resolved after about 15 days and, soon after that, redness of OD appeared. Unspecified eye drops application was useless. On ocular examination, the patient had a Best Correct Visual Acuity (BCVA) of perception of light (PL) in OD with an intraocular pressure of 27 mmHg and obvious proptosis. Visual acuity was normal in the left eye. Slit lamp examination documented a conjunctival hyperemia and conjunctival injection, with diffuse fine keratic precipitates (KPs) distributed on the corneal endothelium, anterior chamber inflammation graded as 1+ cells according to SUN classification [13], irido‐lenticular synechiae, and poorly reacting pupils. On fundoscopy, the retina was not visualizable due to severe vitritis, graded as 4+ according to the National Eye Institute (NEI) vitreous haze scale [14]. The A‐scan and B‐scan ocular ultrasound imaging documented medium‐high reflectivity echoes in vitreous chamber and infero‐nasal medium‐high reflectivity mass projecting into the vitreous (Figure 1). The diagnosis of panuveitis was made.

**Figure 2:**
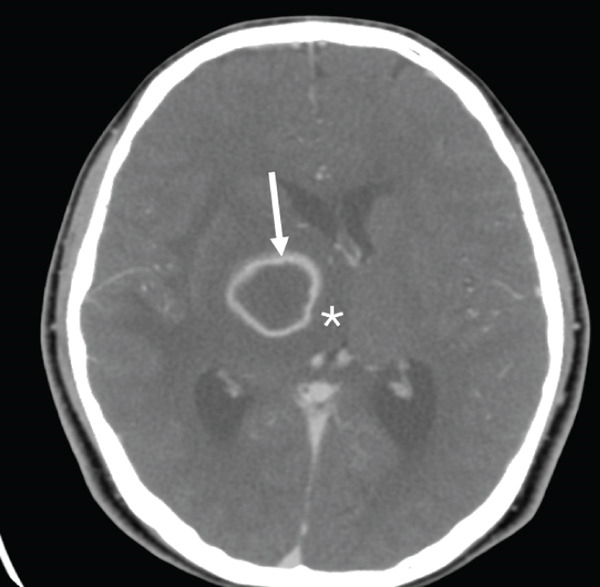
The brain CT image showed a lesion with central necrotic‐colliquative area, surrounded by an external capsule (arrow) and edema (asterisk).

The brain computed tomography (CT) showed a round lesion of 25 × 27 mm in the right hemisphere, between the thalamus, the capsule, and the basal ganglia, with a central necrotic‐colliquative area, surrounded by an external capsule (with contrast enhancement) and edema causing compression and a 7‐mm shift of the cerebral midline (Figure 2). There was also a thickening of the right choroid and retina.

**Figure 3:**
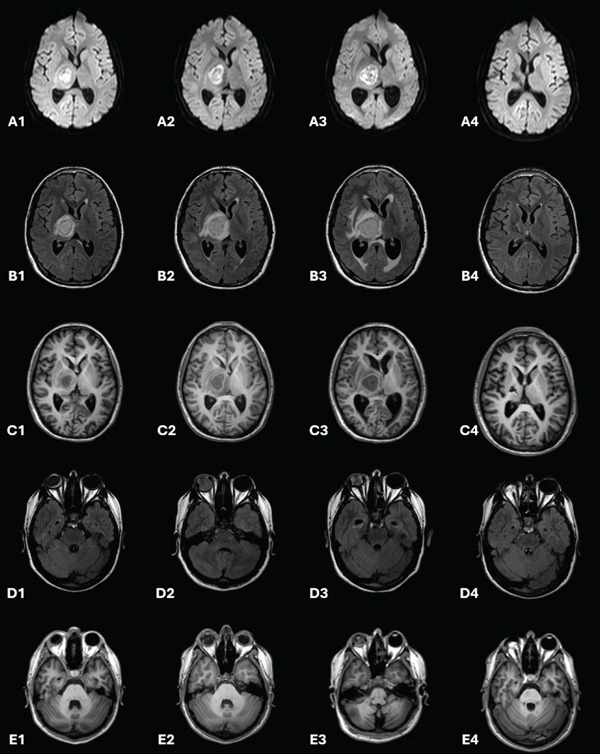
The figure illustrates different magnetic resonance imaging (MRI) acquisition sequences: diffusion‐weighted imaging (DWI) (row A), fluid‐attenuated inversion recovery (FLAIR) (rows B and D), and T1‐weighted imaging (rows C and E). Columns 1–4 represent four different acquisition time points, corresponding to sequential MRI examinations performed over time. A1–E1 show two different sections of the first MRI. A1–C1: the images show a lesion in the right cerebral hemisphere with central necrotic‐colliquative area, surrounded by an external capsule and perilesional edema. D1–E1: the images show intraocular inflammation with mass, projecting into the vitreous. A2–E2 show the third MRI 1 month later. A2–C2: enlargement of the lesion on the right hemisphere with persistence of ring enhancement and millimetric median line shift. D2–E2: funnel‐shaped retinal detachment. A3–E3 show the MRI performed 3 months later. A3–C3: enlargement of the tuberculoma with increased mass effect. D3–E3: right eye with phthisis bulbi. A4–E4 show the last MRI performed 16 months later after neurosurgical intervention (A4–C4) and enucleation (D4–E4).

Neurological examination showed a slight right body motility deficit, bilateral positive Hoffmann sign with left prevalence, normal cerebellar function and reflexes, rare clones of knees and left foot after mobilization. Brain magnetic resonance imaging (MRI) demonstrated a ring‐enhancing lesion within the right thalamo‐capsular region with surrounding edema, mass effect, and estimated an 8–9 mm shift, while orbital MRI confirmed marked inflammation within the eye consistent with endophthalmitis (Figure 3A1–E1). A neurosurgical approach was not recommended.

The case was submitted to the infectious diseases consultant who suspected cerebral toxoplasmosis and put the patient on trimethoprim/sulfamethoxazole 80/400 mg, 2.5 vials every 6 h for an estimated weight of 85 kg. Lumbar puncture was not performed due to the mass effect of the lesion. To investigate the patient immune status, the team performed an HIV test (negative) and lymphocyte subpopulations and serum immunoglobulins, which resulted to normal.

The ophthalmology team performed a diagnostic 25‐Gauge (G) vitrectomy on September 9th, with aspiration of approximately 10 mL of vitreous fluid. The sample tested negative for *Toxoplasma gondii*, CMV, *M. tuberculosis*, bacteria, and fungi. Toxoplasma, Bartonella, and Borrelia serologies were also negative.

The IGRA showed TB1–Nil = 7.340 IU/mL and TB2–Nil = 7.340 IU/mL (cutoff ≥ 0.35 IU/mL), with adequate mitogen response and no history of TB vaccination, so the patient was transferred to the Infectious Diseases ward for suspected cerebral and ocular TB. Here, the patient started specific therapy with isoniazid 300 mg/day, pyrazinamide 1500 mg/day, rifampin 600 mg/day, ethambutol 1200 mg/day, and linezolid 600 mg/day—the latter to enhance brain and ocular penetration—and dexamethasone according to national and international guidelines [15, 16].

Although the chest X‐ray did not show any signs of pulmonary TB, the CT scan highlighted a single nodule in the posterior‐basal segment of the left lung and multiple enlarged lymph nodes in the mediastinum, right hilar, and epiphrenic regions. The abdomen and bone were normal. Blood cultures, urine cultures, gastric aspirate, and stool specimens were all negative for *M. tuberculosis.* As a consequence, because of the strong clinical and radiological suspicion of TB, a second vitrectomy was performed on September 13th. In this sample, *M. tuberculosis* DNA was detected—even if in very low quantity so that rifampin resistance genotypic test could not be performed. The pathological exam showed features of endophtalmitis. The following brain CT and MRI showed progressive improvement.

The patient was discharged on October 10th and continued his follow‐up in the outpatient clinic. The follow‐up MRI detected an enlargement of the lesion on the right hemisphere with persistence of ring enhancement and millimetric median line shift and a retinal detachment in the right eye (Figure 3A2–E2); therefore, the patient was admitted again in November to the Infectious Diseases ward where he underwent a brain biopsy to confirm the diagnosis of disseminated TB. A third 25‐G vitrectomy was performed for retinal detachment and proliferative vitreoretinopathy. Microscopic research after Ziehl–Neelsen coloring resulted intensively positive for acid‐fast bacilli (semi‐quantitative evaluation was ++++) and so were *M. tuberculosis* complex DNA on Gene xpert cepheid, MTB/RIF ULTRA. After the due incubation period, even culture resulted positive for drug‐sensitive *M. tuberculosis* both on solid culture system Löwenstein–Jensen and liquid culture system Mycobacteria Growth Indicator Tube (MGIT) Modified Middlebrook 7H9.

The standard treatment regimen with isoniazid, rifampicin, pyrazinamide, and ethambutol (HREZ) was confirmed while linezolid was discontinued on November 21st after 70 days of five‐drug therapy and after obtaining the resistance tests results. As a consequence of a further enlargement of the brain tuberculoma (34 mm vs. 30 mm) with increased mass effect detected by a follow–up MRI performed on December 21st (Figure 3A3–E3), the patient underwent a neurosurgical intervention to remove this lesion. Microscopic (+−−−), molecular, and cultural microbiologic tests were still positive for *M. tuberculosis* even after 149 days of effective treatment. Histologic and cytologic examination confirmed the tubercular abscess nature of the lesion.

Surgical intervention was complicated by left hemiparesis. An intense rehabilitation treatment was started soon after the intervention and continued in a rehabilitation facility. The patients slowly recovered and partially regained the quo ante condition in both the upper and lower left limb.

He is still followed in outpatient care and he has continued the planned 12‐months antibiotic therapy for cerebral TB starting again after the brain surgery until January 30th, 2026 (Figure 3A4–C4).

**Figure 4:**
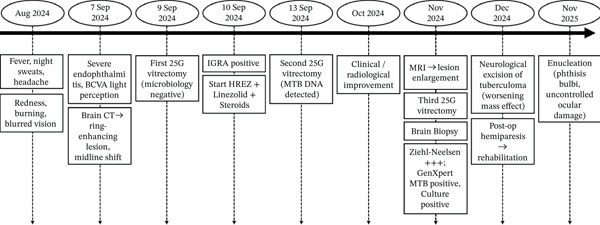
Chronology of systemic symptoms, ocular involvement, neuroimaging findings, microbiological confirmation, therapeutic interventions, and surgical procedures from initial presentation to long‐term outcome in a pediatric patient with disseminated *Mycobacterium tuberculosis* infection involving the eye and central nervous system.

In November 2025, enucleation with autologous dermis fat graft was performed, due to uncontrolled ocular hypertonicity, phthysis bulbi, and visual residual (uncertain light perception) (Figure 3D4,E4) (Figure 4).

## 3. Discussion

This case illustrates the aggressive and multifaceted presentation of extrapulmonary TB involving both the eye and the CNS in a pediatric patient. Pediatric ocular TB is an uncommon manifestation of *M. tuberculosis* infection and remains challenging to diagnose due to its nonspecific clinical presentation. Reported pediatric cases are limited, but most describe posterior segment involvement such as choroiditis, multifocal choroidal lesions, and uveitis, often in the context of disseminated or miliary TB [5]. Diagnosis is frequently indirect, relying on systemic evaluation and response to antitubercular therapy rather than microbiological confirmation from ocular samples. Concomitant CNS and ocular TB has been described only in isolated pediatric case reports and small series. These cases likely reflect hematogenous dissemination and are often associated with tuberculous meningitis or intracranial tuberculomas. Importantly, ocular findings may be subtle or initially overlooked, and neurological manifestations frequently dominate the clinical picture, delaying ophthalmologic diagnosis [17, 18]. Early multidisciplinary assessment is therefore essential, as recognition of ocular involvement may support systemic diagnosis and guide timely treatment initiation. Ocular TB, particularly when presenting as endogenous endophthalmitis or panophthalmitis, is a rare but well‐documented manifestation of *M. tuberculosis* infection. In a systematic review of endogenous tuberculous endophthalmitis and panophthalmitis, pediatric patients accounted for approximately one quarter of reported cases, indicating that severe ocular involvement can occur even in childhood and often reflects disseminated disease rather than isolated ocular infection [19]. According to this review, tuberculous endophthalmitis and panophthalmitis most presented as unilateral disease with rapid progression. Despite antitubercular treatment, the visual prognosis was poor, with more than 80% of reported cases ultimately requiring enucleation, evisceration, or orbital exenteration. Similarly, despite repeated pars plana vitrectomies, prolonged antitubercular therapy, and corticosteroid treatment, our patient developed phthisis bulbi and ultimately underwent enucleation. This further supports the concept that pediatric ocular TB may represent part of a broader systemic involvement, frequently including CNS dissemination, and underscores the importance of early recognition of ocular signs in the context of multisystem TB.

At presentation, before the availability of IGRA results and vitreous investigations, the patient exhibited a severe unilateral panuveitis characterized by dense vitritis (NEI grade 4+), markedly reduced visual acuity, and an obscured fundus, together with a cerebral ring‐enhancing lesion with central necrosis. Based on these findings, the differential diagnosis included ocular toxoplasmosis, ocular TB, viral retinitis, endogenous endophthalmitis, and less likely, noninfectious inflammatory disorders. This differential is consistent with the typical etiological spectrum of severe infectious posterior uveitis [20]. Ocular toxoplasmosis was considered a relevant diagnostic hypothesis because it represents the most common cause of infectious posterior uveitis worldwide and may present with severe vitritis, sometimes precluding fundus visualization [20, 21]. Furthermore, although uncommon in immunocompetent individuals, cerebral toxoplasmosis has been reported and therefore could not be excluded in the presence of a concomitant necrotic brain lesion [22]. Ocular TB was also included in the differential diagnosis, as it represents an important cause of infectious posterior uveitis with highly variable clinical manifestations and often requires microbiological and immunological confirmation [5]. However, at that stage, no laboratory evidence supporting TB was yet available, and chest radiography did not reveal findings suggestive of pulmonary TB. Given the severity of the ocular presentation and the risk of irreversible visual loss, empiric treatment with trimethoprim/sulfamethoxazole was initiated while awaiting the results of IGRA testing and vitreous analysis.

The incidence of tubercular uveitis differs geographically and correlates with the regional prevalence of TB. It is estimated at approximately 3% in non‐endemic countries, increasing to about 7% in endemic areas [12, 23]. The absence of pulmonary involvement and the initial negativity of microbiological tests further complicate the diagnostic process. Our experience underscores the need for a high index of suspicion for TB in patients with severe intraocular inflammation and compatible epidemiological exposure, even in the absence of systemic findings. Repeated invasive diagnostic procedures may be required to obtain microbiological confirmation. The progressive enlargement of the tuberculoma and the worsening of the eye features could be reconducted to a paradoxical Immune Reconstitution Inflammatory Syndrome (IRIS) that occurs during TB treatment and has been attributed to the release of new antigen targets during mycobacterial killing, hypersensitivity to such antigens, and exaggerated immune restoration (following TB‐induced immunosuppression) [24]. The case highlights the importance of a multidisciplinary team to manage neurological and ocular complications in this context, alongside steroids. Management of such cases requires a coordinated multidisciplinary approach involving ophthalmology, infectious diseases, neuroradiology, and neurosurgery. Early recognition and timely initiation of targeted therapy are crucial to reduce irreversible ocular damage and long‐term neurological sequelae. This report reinforces the importance of considering TB in the differential diagnosis of severe uveitis and endophthalmitis, particularly in young patients from endemic areas.

**Nomenclature**
BCVA: best correct visual acuity
CNS: central nervous system
CT: computed tomography
G: gauge
HREZ: isoniazid, rifampicin, pyrazinamide and ethambutol
KP: keratic precipitate
MRI: magnetic resonance imaging
OD: right eye
PL: perception of light
TB: tuberculosis

## Author Contributions

N.D.S. and L.C. were responsible for conceptualization, data curation, investigation, methodology, visualization, writing – original draft and writing – review and editing. E.B. was responsible for data curation, investigation and writing – original draft. B.I. and C.P. were responsible for investigation and methodology. D.R. and T.F. were responsible for supervision and validation. G.V.L.D.S. and C.C. were responsible for writing – review and editing. N.D.S. and L.C. contributed equally to the case report.

## Funding

No funding was received for this manuscript. Open access publishing facilitated by Universita degli Studi di Perugia, as part of the Wiley ‐ CRUI‐CARE agreement.

## Consent

Written informed consent for publication of this case report and accompanying images was obtained from the patient’s parents.

## Conflicts of Interest

The authors declare no conflicts of interest.

## Data Availability

The clinical and imaging data used to support the findings of this study are included within the article. Additional details are available from the corresponding author upon reasonable request, subject to ethical and privacy considerations.
